# Cytomegalovirus Seroprevalence in Northern Poland in the Population Planning Pregnancy and Pregnant Women

**DOI:** 10.3390/v17040537

**Published:** 2025-04-07

**Authors:** Małgorzata Świątkowska-Freund, Szymon Bednarek, Natalia Sasak-Cieślar, Natalia Kocięcka, Paweł Powroźnik, Anna Waldman

**Affiliations:** 1 The Academy of Applied Medical and Social Sciences, Lotnicza 2, 82-300 Elbląg, Poland; 2 The Ludwik Rydygier Provincial Polyclinical Hospital in Torun, Św. Józefa 53-59, 87-100 Toruń, Poland; prenatalium@gmail.com (S.B.); natalia.sasak@gmail.com (N.S.-C.); 3 Powiatowe Centrum Zdrowia w Kartuzach, Ceynowy 7, 83-300 Kartuzy, Poland; nataliakociecka@gmail.com; 4 Medical University of Gdansk, Marii Skłodowskiej-Curie 3a, 80-210 Gdańsk, Poland; pawelpow2@gmail.com; 5 Poznan University of Medical Sciences, Collegium Maius, Fredry 10, 61-701 Poznań, Poland; anna.waldman29@gmail.com

**Keywords:** cytomegalovirus, seroprevalence, pregnancy

## Abstract

Cytomegalovirus is an enveloped DNA virus. All forms of CMV infection—primary infection, reactivation, and infection with a different strain—may be asymptomatic. The risk of vertical transmission in the periconceptional period is approximately 20%, the risk of primary infection in the first trimester is approximately 30%, and in the third trimester the risk increases to 70%. However, the most severe forms of congenital cytomegaly in newborns are related to infections in the periconceptional period. Offering a vaccine to the seronegative patients planning pregnancy may decrease incidents of congenital cytomegaly in neonates. The authors performed retrospective analysis of seroprevalence of CMV in 909 women who reported for pre-conceptional visits or routine pregnancy follow-ups (2003–2023). In the analyzed group, 577 (63.7%) women were seropositive. No influence related to the women’s age and place of residence was found. Higher seroprevalence was observed in women with children or those working in contact with many people. In the group of 332 seronegative patients, 21 (0.6%) were diagnosed with primary infection during pregnancy. Vaccinating 36.3% of patients planning pregnancy could significantly decrease the risk of primary infection during pregnancy, vertical transmission of CMV, and symptomatic infection in the neonates.

## 1. Introduction

Cytomegalovirus (CMV) is an enveloped DNA virus, similar to other members of the herpes virus family. CMV infection is spread through contact with contaminated bodily secretions (such as urine, saliva, genital secretions, and breast milk) and generally causes no or few symptoms in immunocompetent individuals, but can cause serious damage in immunosuppressed ones, including fetuses [1,2,3].

Vertical transmission can occur during the primary infection, the reactivation of the disease, or even due to contamination with another strain [2]. Sensorineural hearing loss, cerebral palsy, developmental delay, epilepsia, infectious eye diseases, and icterus are the most frequent congenital cytomegaly manifestations. Severe complications, like microcephaly, growth restriction, resulting in intrauterine death, are also possible [2,3,4,5,6].

The risk of vertical transmission after primary infection in pregnant woman in the first trimester is approximately 30%, and in the third trimester this increases up to 70% (the highest rate of transmission through the placenta). However, the most severe forms of congenital cytomegaly in newborns are related to infections in the periconceptional period (which is up to 4 weeks before or 3 weeks after a missed menstrual period) [4,7]. After primary infection in the second and third trimesters, according to some authors, the symptoms are not visible in children. According to others, these are visible but milder and may include hearing loss (usually one-sided) diagnosed in childhood [8]. The risk of materno-fetal transmission during reactivation and non-primary infection is much lower and less harmful for the fetus [9]. Despite the belief that children of women with primary CMV infection during pregnancy have the highest risk of vertical transmission, in many countries they are not the biggest group among all congenital CMV infections, because seropositive women are much more frequent than seronegative women. Even in countries with relatively low seroprevalence, many children may be infected during the mother’s reactivation or reinfection [9].

Most infected newborns are asymptomatic, but 15% of them may manifest symptoms of congenital cytomegaly [1,10,11]. Diagnosing asymptomatic CMV infection during pregnancy is very helpful, specifically early diagnosis, which enables the proper monitoring and treatment of the child.

Reducing the risk of infection (primary prophylaxis) is based on maintaining hygiene rules by seronegative individuals, in which the cytomegalovirus infection may have a severe course. This group includes pregnant women, and thus indirectly the fetuses. Vaccine candidates are under investigation [6,10]. As for now, secondary prophylaxis is possible, decreasing the risk of vertical transmission (valacyclovir, hyperimmunized immunoglobulin) and treatment of the fetuses with prenatally diagnosed symptomatic cytomegaly [4,5].

Seroprevalence is the percentage of people in a population who have antibodies in their blood that show they have been exposed to a virus or other infectious agents. Studying the seroprevalence of antibodies in a specific virus can show how many people have been infected with that virus [12], or conversely, how many of them are susceptible to the infection and may benefit from vaccination.

One published paper reported that the global prevalence of CMV is 83% in the general population, 86% in women of childbearing age, and 86% in blood or organ donors. The study also showed that the prevalence is higher in lower socioeconomic groups [1]. These data are important because the estimated seroconversion in pregnant women is higher than the prevalence of CMV in the general population.

Knowing the seroprevalence rate in a population is very important for choosing the best strategy for screening and treating congenital CMV. If the seroprevalence is intermediate (e.g. 50% in Western Europe), half of congenital infections are due to a primary infection during pregnancy, and it is worth screening at the beginning of pregnancy and then treating with valaciclovir. If the seroprevalence is high (e.g., over 90% in Brazil), most congenital infections are due to reactivations or reinfections during pregnancy. It is then much more useful to carry out systematic neonatal screening and search for CMV in the amniotic fluid in the event of signs in a prenatal ultrasound. Authors refer to national population data because the study presented below refers to seroprevalence in Poland.

The first recommendations regarding CMV serological screening during pregnancy were published in 2024. Testing for CMV is advised to be carried out every 4 weeks until 16 weeks of gestation [5]. Whether screening is economically justified is still debated, but pre-conceptional vaccination would certainly be the best solution for the congenital CMV problem.

The authors retrospectively analyzed CMV seroprevalence in the population planning pregnancy and in pregnant women in northern Poland in 2003–2023.

## 2. Materials and Methods

The authors performed a retrospective analysis of seroprevalence of CMV in Polish women at reproductive age. Women who reported for a pre-conceptional visit or routine pregnancy follow-up to the Pomeranian Center of Prenatal Diagnosis and Therapy (Gdansk, Pomeranian region) and Our Doctor Center in Torun (Kujavian-Pomeranian region) in 2003–2023 were routinely advised to perform serological tests for cytomegalovirus—the IgG and IgM antibodies. The tests were not obligatory, and they were financed by patients. The schedule of testing during pregnancy was based on the literature available at this time.

Patients presented with test results were advised as follows.

1. Before pregnancy:
  - Negative IgM and positive IgG; no testing after conception.
  - Negative IgM and IgG; repeated testing in the first trimester, after 20 and after 30 weeks of gestation.
2. Women who were not tested before pregnancy were then tested for the first time during pregnancy:
  - Negative IgM and positive IgG; IgG avidity was scheduled—in cases with high avidity testing was not repeated, patients with low avidity were referred for further testing (PCR, repeated serology).
  - Positive IgM and IgG; IgG avidity was scheduled, patients with low avidity were referred for treatment, patients with high avidity were referred for further testing (PCR, repeated serology).
  - Negative IgM and IgG—repeated testing after 20 and after 30 weeks of gestation.

Avidity was defined as the aggregate strength with which a mixture of polyclonal IgG molecules binds to multiple antigenic epitopes of proteins (in our study, the analyzed test format was an enzyme-linked immunosorbent assay (ELISA)) [13]. There were no patients with intermediate avidity. Patients performed tests in different laboratories, every laboratory had its own referral ranges describing positive and negative results and high and low avidity. The results were interpreted according to the referral ranges of every laboratory.

The data were collected in Excel Sheets. The authors collected any data that may have a potential influence on the risk of being seropositive: patient’s age, having at least one child, type of work (contact with children—teachers, pediatric professionals, daycare workers or large numbers of adults—physicians, nurses, clerks, shop assistants), inhabitance (village, small city with population up to 100,000, big city with a population over 100,000). Then, the results of the laboratory tests were collected: IgG, IgM, and avidity. The seroprevalence of cytomegalovirus was analyzed in relation to parity, type of work, and place of residence.

Statistical analysis was based on Shapiro–Wilk, Levene, Anova, Kruskal–Wallis, χ^2^, *t*-Student, and Mann–Whitney tests, and the significance threshold was *p* < 0.05.

## 3. Results

The serologic status of 909 patients was analyzed. The median age of the patients was 31 years (17 to 50). In total, 155 (17.3%) lived in villages, 96 (10.7%) in small towns (up to 100,000 inhabitants), and 644 (72.0%) in big cities.

At least one child was born before the first cytomegalovirus test for 416 (45.8%) women. In this group, 294 (70.7%) had one child, the rest of them at least two.

The authors analyzed the occupation of the women. A total of 268 (41.0%) patients reported a lot of contact with children in work, 322 (49.2%) had contact with many adults and 64 (9.8%) had contact with no or a very limited number of people.

During the testing, 283 (31.1%) women performed the first test before pregnancy, and the remaining 626 (68.9%) were tested for the first time during pregnancy.

In the analyzed group, 577 (63.7%) women were seropositive at the first test. There was no difference between women tested for the first time before or during pregnancy (61.5% and 64.7%, respectively; *p* = 0.104, *t*-Student test).

The influence of age, residence, having children, and type of work on the seroprevalence was analyzed.

The age of the patient did not show any influence—seroprevalence was the same in all age groups (*p* = 0.328, U Mann–Whitney test). No influence of place of residence was found (*p* = 0.828%, Kruskal–Wallis test). Women with at least one child had higher seroprevalence than women with no children (*p* < 0.001, Kruskal–Wallis test). The authors did not analyze if having one or more children influenced the seroprevalence in the given analyzed group.

Patients working with no, or with very limited, contact with people had lower seroprevalence than women who had contact with people, including children and adults (*p* = 0.043, Kruskal–Wallis test). The data are presented in Table 1.

In the analyzed group, 21 primary cytomegalovirus infections during pregnancy were diagnosed, with the majority of them being during the first trimester (14 during the first and 7 in the second trimester). Only 0.6% of women were seronegative. We did not have information regarding children from all the patients in the study group, and the correlation between the primary infection and having children has to be interpreted carefully. In the primary infection group, 14 women had no children, which means that the risk of infection seems to be higher in nulliparous (2.8%) than in multiparous women 1.7%).

Three women with primary infection during pregnancy were diagnosed after 2020, when valacyclovir was used as cytomegalovirus secondary prevention in our center. In one patient, vertical transmission was confirmed (patient infected in the second trimester and treated with valacyclovir) in an amniocentesis performed 7 weeks after primary infection. All children are healthy, with no symptoms of congenital cytomegaly.

## 4. Discussion

The cytomegalovirus infection during pregnancy is a serious health hazard and a socioeconomic problem. It is the most frequent congenital pathology caused by infection [2,4]. The spectrum of complications includes severe permanent disability. A total of 2.5% of neonates are born with congenital cytomegaly, and one in every five then may present with significant health problems [4,5]. Testing women before and during pregnancy may reveal asymptomatic maternal infection and enable primary prophylaxis, including vaccination, secondary prophylaxis, and early treatment.

The possibility of infection during pregnancy is a problem faced in all countries, including highly developed ones. Women whose children attend group facilities (e.g., preschool) and who work with children, such as in pediatric health care institutions, are at high risk of infection during gestation [4].

In 2019, Zuhair et al. reported an 83% cytomegalovirus prevalence, calculated for the global population. The highest percentage was observed in mediterranean countries (Turkey,92%) and the lowest in Europe (Ireland, 44%). Regardless of the region, higher seroprevalence was found in women of childbearing age (mean 86%) and seemed to increase with age, but these data were limited. The Polish population’s prevalence was placed in the median value, resulting in a little over 70% [5]. In a paper published in 2016, it was concluded that Polish women’s seroprevalence was related to their age, and was at approximately 82%, but their study group was relatively small [3,14].

The presented study covered the Pomeranian and Kujavian-Pomeranian regions, where seroprevalence was 63.7%, which was lower when compared to a study performed 10 years earlier in central Poland, where the prevalence was 76.7% [15]. The authors of the paper analyzed seroprevalence from 1999 to 2009 and noted a decrease in seroprevalence with time. The results we presented above confirmed this trend. This may be related to an improvement in the socioeconomic status of the patients and a higher awareness of hygiene rules. It is also possible that lifestyle changes, leading to the decreasing popularity of group facilities for small children in Poland and more children remaining at home with family during workdays, have had an impact on the frequency of asymptomatic infections in women.

It has been reported that 50% of seronegative mothers are infected during the first 2 years of life of their first child [2]. This suggests that multiparas should be more frequently seropositive and have lower risk of primary infection during pregnancy. In our material, this hypothesis was confirmed. In primiparas, the frequency of primary infection was 2.8% and in multiparas, it was 1.7%.

In the analyzed group, the correlation of seroprevalence with the age of women was not confirmed. It was observed by Siennicka and Flanders, and was suggested by Zuhair [3,9,14]. The correlation between positive IgG and having children the presented study was significant. The literature proves that women, specifically those of childbearing age and with children, have higher seroprevalence than men. This is related to becoming infected by children during their first years of life, as women more frequently take care of sick children [4,16].

The act of taking care of small children at home (family, baby-sitters) and in facilities for children (preschools) is usually performed by women. This is probably one of the causes of higher seroprevalence in woman when compared to men [4,16]. In our study population, a tendency towards higher seroprevalence in a group of women who had contact with many children at work was observed.

CMV screening is now being discussed in Poland. Low seroprevalence population benefits more from prenatal screening, and in populations of high seroprevalence, neonatal screening that detects asymptomatic congenital cytomegaly is more useful. Within the Polish population, like in other Western Europe countries, seroprevalence is intermediate. Prenatal screening enables the detection of primary infections in early pregnancies and the prevention of congenital cytomegaly. Postnatal screening of neonates detects asymptomatic infections in a group of seropositive mothers and enables adequate treatment of the children to prevent hearing loss and other complications. According to the opinions of the authors of the study, ideally, prenatal screening in seronegative patients should be performed, with an option of postnatal screening for children of seropositive mothers. 

The study was performed in two outpatient facilities, both in big cities. A total of 28% of the study group lived in small cities and villages, and this is not representative of the Polish population, as only 13.7% live in big cities. Seroprevalence was similar for both groups, and it should not influence the results. 

## 5. Conclusions

Testing for CMV antibodies in women of childbearing age enables the identification of the group at high risk of primary CMV infection during pregnancy. Decreasing seroprevalence increases this group. The possibility of vaccination of seronegative women would enable more effective primary prevention of congenital CMV.

Despite there being no recommendations regarding regular screening during pregnancy of seronegative women in Poland [16] and most other countries, suggestions to perform it every four weeks until 16 weeks of gestation seems reasonable [6]. It is under discussion, if testing for primary infection later in pregnancy and testing of seropositive asymptomatic women should be offered to patients. Seronegative women after seroconversion are offered secondary prophylaxis, and maybe in the near future these options in reinfections and reactivations of CMV infection will also be considered.

## Figures and Tables

**Table 1:** Cytomegalovirus seroprevalence in relation to maternal age, place of residence, parity, and contact with people.

		Seropositive	Seronegative	*p* Value
Maternal age (years)		31	31	0.328
Place of residence	Village	104 (67%)	51 (33%)	0.828
	Small cities	64 (67%)	32 (33%)	
	Big cities	411 (64%)	233 (36%)	
Parity	At least one child	277 (67%)	139 (33%)	<0.001
	No children	302 (61%)	191 (39%)	
Contact with people	Occasional	39 (61%)	25 (39%)	0.043
	With children	221 (65%)	101 (35%)	
	With adults	174 (69%)	94 (31%)	
Total		577 (64%)	332 (36%)	

## Data Availability

Data available on request from the authors.

## Abbreviations

The following abbreviations are used in this manuscript:

CMV	Cytomegalovirus
